# Internet treatment for social anxiety disorder in Romania: study protocol for a randomized controlled trial

**DOI:** 10.1186/1745-6215-13-202

**Published:** 2012-10-30

**Authors:** Bogdan Tudor Tulbure, Kristoffer NT Månsson, Gerhard Andersson

**Affiliations:** 1 Department of Clinical Psychology and Psychotherapy, Babes-Bolyai University, No 37 Republici Street, Cluj-Napoca, 400015, Romania; 2 Department of Behavioral Sciences and Learning, Linköping University, Linköping, SE-581 83, Sweden; 3 Department of Clinical Neuroscience, Karolinska Institutet, Karolinska University Hospital Huddinge, Stockholm, SE-141 86, Sweden

**Keywords:** Internet-administrated cognitive behavior therapy, Social anxiety disorder, Social phobia, Guided self-help, Randomized controlled trial

## Abstract

**Background:**

Social anxiety disorder (SAD) is one of the most common anxiety disorders and is associated with marked impairments. However, a small proportion of individuals with SAD seek and receive treatment. Internet-administrated cognitive behavior therapy (iCBT) has been found to be an effective treatment for SAD. This trial will be the first Internet-delivered guided self-help intervention for SAD in Romania.

**Methods:**

Participants with social anxiety disorder (*N* = 96) will be recruited via newspapers, online banners and Facebook. Participants will be randomized to either: a) an active treatment, or b) a waiting list control group.

The treatment will have a guided iCBT format and will last for nine weeks. Self-report questionnaires on social phobia, anxiety, depression, treatment credibility and irrational thinking will be used. All assessments will be collected pre, post and at follow-up (six months after intervention). Liebowitz Social Anxiety Scale – Self-Report version (LSAS-SR) will be the primary outcome measure and will be administrated on a weekly basis in both conditions.

**Discussion:**

The present randomized controlled trial investigates the efficacy of an Internet-administered intervention in reducing social anxiety symptoms in a culture where this form of treatment has not been tested. This trial will add to the body of knowledge on the efficacy of iCBT, and the results might lead to an increase of the accessibility of evidence-based psychological treatment in Romania.

**Trial registration:**

ClinicalTrials.gov: NCT01557894

## Background

Social anxiety disorder (SAD) affects 6.5% of Europeans (for lifetime prevalence, see [1]), and is associated with marked impairments in personal, professional, and social life. SAD is associated with higher rates of divorce, alcohol consumption, suicide risk, and other forms of psychopathology [1,2]. Everyday functioning is significantly altered, and greater financial dependence on family and/or state resources makes SAD a societal burden [2]. Epidemiological data also reveal that only a small number of individuals seek and receive treatment for this condition [1], as SAD sufferers can delay seeking treatment for 28 years [3]. Spontaneous remission of symptoms has been reported and is rare, and it is mainly by active interventions (psychological and pharmacological) that the course of SAD can be altered [4].

The Internet has grown rapidly across the world and the number of users increases steadily ( http://www.internetworldstats.com). An association between SAD and Internet use has been found [5,6], suggesting that socially anxious individuals use the Internet to search for information and to communicate with others. Moreover, communication via the Internet may be attractive to socially anxious individuals because it conceals physical appearance and aspects of behavior that in face-to-face interactions could be viewed as being negatively evaluated by others [5].

Given that the core features of SAD are the fear and avoidance of direct social interactions, guided Internet-administrated cognitive behavior therapy (iCBT) may constitute an attractive treatment option for persons with SAD [7]. These individuals may be drawn to such treatment because they can engage in the therapeutic process from their private homes or offices and reduce the embarrassment and stigma of seeking treatment and going to a therapist’s office. However, there is a potential risk that isolation and avoidance is reinforced by not seeing the client in therapy. To counteract this propensity, iCBT for SAD includes not only cognitive restructuring sessions, but also real life exposure exercises where participants are encouraged to confront their fears in their daily life [8].

Andersson *et al*. [9] were the first to test the efficacy of an iCBT for SAD. To maximize the effects, the nine online modules were augmented with two therapist-lead group exposure sessions. Treated individuals reduced their social anxiety symptoms, depression and general anxiety, showing clinical significant changes [9]. Since then, other Internet-delivered interventions for SAD have been developed and tested in Australia, Spain, Sweden and Switzerland [10-17], supporting the short- and long-term benefits of such interventions. In a meta-analysis of eight studies, the overall efficacy of iCBT for SAD was found to be high for social anxiety symptoms (Cohen’s *d* test statistic = 0.86), and moderate for quality of life (*d* = 0.53) and comorbid anxiety and depression (*d* = 0.40) [18]. Recently Hedman *et al*. [19] investigated the cost-effectiveness of a guided iCBT for SAD and a parallel group cognitive behavioral therapy (GCBT) intervention. While both treatments generated large cost reductions, the iCBT intervention was significantly less costly. This difference was mainly attributed to the therapist time required for each participant per week for the two interventions (that is., 5.5 minutes for iCBT versus 50.0 minutes for group CBT per week per patient). Because the savings generated by each treatment exceed their costs, Hedman *et al*. concluded that “society as a whole would be financially strengthened by making CBT for SAD more accessible” [19] (p. 735). The new generation of self-help interventions delivered online represents a readily accessible and cost-effective avenue to increase treatment accessibility for SAD. Nevertheless, before including them in the evidence-based armamentarium, we should carefully investigate their efficacy in various contexts and cultures.

This study represents an extension and replication of previous trials examining the effectiveness of guided iCBT for individuals with SAD. To our knowledge, there are no published trials on iCBT for Romanian adults with SAD. To date, paxonline.ro is the only platform offering computer-assisted intervention for anxiety in Romania [20]. This password-protected website offers online assessment, resources and interventions for patients with different forms of anxiety, but no controlled trials about its effectiveness have yet been published.

In our study, an individual treatment approach will be used not only because the active intervention is delivered online, but also because it has been argued that individual treatments may be superior to group treatments [21]. Consequently, the primary aim of this study will be to investigate the efficacy of a treatment program called internet social phobia (iSOFIE) by comparing it to a waiting list control group. iSOFIE was derived from a previously tested Swedish program [22] that has been shortened while retaining the main treatment ingredients. The program assists participants in understanding their condition, in restructuring their maladaptative thoughts, and in implementing the exposure exercises (i.e., which is known to be a key factor in symptoms reduction) [8]. A second aim of the study is to track the potential changes in participants’ thought patterns. While our intervention is primarily based on the cognitive- behavioral model of SAD [23], and no specific Rational Emotive Behavior Therapy (REBT), [24]), will be implemented, it is hypothesized that the cognitive restructuring and exposure exercises will make participants less prone to irrational thinking - (as measured by the Attitude and Belief Scale-II (ABS-II), [25]). To our knowledge, no study has investigated whether an iCBT program for SAD has the potential to alter participants’ irrational thinking patterns. Moreover, because this is the first guided iCBT interventions conducted in Romania, the treatment credibility of the program will be investigated. Finally, we will investigate predictors of outcome with a focus on the levels of SAD and associated anxiety symptoms.

## Method/design

### Study design

We will conduct a randomized controlled trial (RCT) with two parallel groups: the iSOFIE and a waiting list control group. The study has been approved by the regional Institutional Review Board (30273 - 02/29/2012) and registered in clinicaltrials.gov (NCT01557894).

### Participants

Participants will be recruited through advertisements in national and local Romanian newspapers, online banners, and the Facebook page specifically designed for this purpose. After the initial screening, all potential participants who score above the cut-off on the Leibowitz Social Anxiety Scale – Self-Report version (LSAS-SR) [26,27], Social Phobia Inventory (SPIN) [28], and Social Interaction and Anxiety Scale (SIAS) [29] will be contacted for a structured telephone interview. The interview will be based on the Structured Clinical Interview (SCID) for of the Diagnostic and Statistical Manual of Mental Disorders - IV (DSM-IV), [30]).

To be included in the study, participants have to fulfill the following criteria:

1. be over 18 years old

2. exceed the cutoff scores on SPIN, SIAS, and LSAS-SR

3. fulfill the DSM criteria for SAD on the Social Phobia Screening Questionnaire (SPSQ)

4. display no suicidal ideation (i.e., not exceed a score of 2 on the suicide item of (BDI-II) and not report parasuicidal behavior on the Screening Questionnaire of the SCID)

5. do not undergo other forms of treatment for SAD

6. have access to a computer connected to the Internet

7. if participants are on medication, the dose should be constant for at least 1 month, and participants should agree to keep the dosage unchanged for the whole time of the study

8. have SAD as the primary diagnosis on the SCID

9. have no diagnosis of psychosis or borderline personality disorder on the SCID

### Statistical analysis

Group differences regarding in demographics and pre-treatment measures will be analyzed with using the chi square (*χ*^2^) test and the independent sample *t*- test. The potential impact of the iSOFIE intervention will be analyzed using analysis of covariance (ANCOVA) or mixed model analysis, followed by the appropriate post-hoc analyses. Effect sizes for both within- and between subject designs will be calculated using Cohen’s *d*-test based on the pooled standard deviation (SD). For the post-treatment data, an intention-to-treat design will be used with appropriate estimation of missing values [31].

### Sample size

The trial will be powered to detect a between- group effect size of *d* = 0.60 at post-treatment (alpha = 0.05, two-tailed) at a power of 80% (1-β). A total of 96 participants will be needed for the study (i.e., that is, *N* = 48 per condition), when considering a potential dropout risk of 20% and intent-to-treat analysis with all included participants in the analyses. With such a sample, we have a reasonable chance to detect the potential statistical and clinically significant effect of the intervention.

### Procedure

Participants will be recruited via newspapers advertisements, a Facebook page created for this purpose, and the project’s website [32]. They will be directed to the project’s website, advised to read the study presentation, and eventually register to participate. Before starting the screening process, interested participants will be advised to carefully read the online Informed Consent details, and to approve their voluntary participation in the trial.

Participants will then receive a code that allows them to access the following screening measures: LSAS-SR, [26,27], SPIN, [28], SIAS, [29], SPSQ, [33], (BDI-II, [34]), (ABS-II), [25,35], and Anxiety Sensitivity Index (ASI), [36]. All these measures are widely used in the literature and have good to excellent psychometric properties. Moreover, for LSAS-SR and SIAS, similar psychometric proprieties were found for both online and paper-and-pencil formats [37]. Participants who score above the cut-off value of the social anxiety measures will be contacted for a telephone interview. In addition, participants’ results on the SPSQ form will also be inspected to see whether they meet the criteria for SAD according to the DSM-IV. The telephone interview is based on social phobia section of the Structural Clinical Interview for DSM-IV - Axis I Disorders: SCID I [30]. Only participants who will meet the inclusion criteria will take part in the study.

Included participants will be randomized by an independent person to either: a) active training (iSOFIE) or b) the waiting list control group. The iSOFIE treatment manual is divided into nine modules and adapted for the Internet. Each module presents a different topic (that is., what is social anxiety, challenging your negative thinking, behavioral experiments, exposure etcetera.) and includes exercises, and essay questions (that is., homework to be sent in to the therapist). Participants are encouraged to explain what they have learned, provide thought records, describe their experiences during the exposure exercises. Based on their responses, an assessment is made of whether the participant is ready to continue. If so, the next module is made accessible. If not, the participant receives instructions on what needs to be completed before proceeding to the next module. The intervention takes nine weeks to be completed. The control group will not receive any active intervention during this time. To monitor participants’ symptoms and to analyze the possible mediating effects, all participants will be asked to fill in the LSAS-SR [27] on a weekly basis.

By the end of the nine-week treatment period, all participants will be asked to complete the post-intervention assessment, which consists of the same measures used in the screening phase, with treatment satisfaction items added for the intervention group. The SAD section of the SCID interview will also be used as a categorical diagnostic measure. To evaluate long-term effects of the intervention, the follow-up data will be collected six months after the treatment. The waiting -list control group will receive treatment after the waiting period and will also be evaluated after they have completed the treatment, and at six months follow-up.

A secure software will facilitate correspondence between the participants and therapists. The online messaging system uses a protocol for encrypting information (Secure Sockets Layer) to ensure communication security over the Internet. All communication will be mediated through this online messaging system.

#### Primary outcome measures

Social anxiety symptoms will be assessed with more than one measure. The LSAS-SR [27] presents 24 commonly anxiety-provoking situations, and asks participants to rate their fear and avoidance for each situation. The psychometric properties of the LSAS-SR are good to excellent [26] and the scale captures symptom changes in both cognitive- behavioral and pharmacological interventions. The LSAS-SR has been recommended as a treatment monitoring, and treatment outcome, evidence-based measure in adults [38] and adolescents [39].

The *Social Phobia Inventory* (SPIN, [28]) is a brief (i.e., 17-item) self-report instrument measuring fear in social situations, avoidance of performance/social events, and physiological discomfort in social situations. Each item is rated on a 4-point scale, with higher scores corresponding to greater distress. The scale has good to excellent psychometric proprieties [28,40] and has been recommended for clinical use [38].

The *Social Interaction and Anxiety Scale* (SIAS, [29]) is a 20- item measure that assesses fears of general social interactions. The scale captures both social scrutiny fears and social interaction fears. For each item, respondents are asked to indicate the degree to which they feel the statement is characteristic or true of them on a five-point scale. The SIAS has sound psychometric properties [29].

The *Social Phobia Screening Questionnaire* (SPSQ, [33]) is a measure used for diagnostic screening of SAD. The questionnaire presents both dimensional and categorical data, including impairment and duration of reported social anxiety [33]. It has been used in several other studies of SAD [9,13,14,41].

#### Secondary outcome measures

In addition to the social anxiety measures, we intend to capture participants’ anxiety sensitivity, depression level, and irrational thinking, using the *Anxiety Sensitivity Index* (ASI, [36]), *Beck Depression Inventory-II* (BDI-II, [34,42,43]), and *Attitude and Belief Scale-II* (ABS-II, [25,35]).

The *Anxiety Sensitivity Index* (ASI, [36]) is a 16-item questionnaire that measures the beliefs about the social and somatic consequences of anxiety symptoms. The total ASI score ranges from 0 to 64, and is the sum of the scores on the 16 items. The scale has sound psychometric properties [36,44,45].

The *Beck Depression Inventory-II* (BDI-II, [43]) is a 21-item self-report inventory widely used to assess DSM-IV depressive symptoms. Each item consists of four statements, scored 0– to 3, indicating increasing symptom severity. Sound psychometric proprieties have been reported for the BDI-II [43].

The *Attitude and Belief Scale-II* (ABS-II, [25]) measures rational and irrational thinking as described by Albert Ellis [24]. The scale was designed to capture four cognitive processes (namely., ‘demandingness’, ‘awfulizing’, ‘low frustration tolerance’, and ‘self-downing/global evaluation’) in three content areas (i.enamely., achievement, approval, and comfort). We selected the 72-item ABS-II because preliminary psychometrics are available for the Romanian population [35].

## Discussion

Reducing social anxiety can have important benefits in terms of both personal and societal costs [1,2]. The present RCT investigates the efficacy of an Internet-administered intervention (iSOFIE) in a different cultural setting. Potential participants will be carefully screened using both self-report measures (namely., the *LSAS-SR, SPIN, SIAS,* SPSQ) and a clinical interview (the SCID for DSM-IV). After the nine-week intervention, all participants (that is., the active treatment group and the waiting list control group) will be asked to complete the post-treatment measures, and the follow-up assessment will be administered after 6 months. Besides capturing the intervention’s effectiveness in reducing social anxiety symptoms, potential changes in participants’ thinking patterns will be estimated. Data about treatment credibility and satisfaction will also be collected. Along with the hypothesized main effects, these data will provide a valuable feedback for optimizing future interventions.

Compared to the relatively new Internet-delivered interventions, face-to-face treatments appear to have stronger empirical support in the literature, and their credibility seems to be higher. However, the effects of the face-to-face treatments were recently compared with the effects of guided self-help for depression and anxiety, and no significant differences were found [46]. It was argued that generalizing the results was hindered by the fact that participants were mainly self-referred (that is., clients interested in guided self-help applied for such trials, and the remaining category of clients applied for the face-to-face treatments). While it is true that some people may benefit more from guided self-help interventions, the fact that both treatments yielded similar effects [46] cannot be discarded. Moreover, investigating the long-term effects of Internet-administered treatment for SAD, Hedman *et al*. [47] found that the improvements in social anxiety symptoms were sustained 5 years after the treatment. This additional evidence supports the idea that guided self-help represents an effective and reliable way of helping anxiety sufferers via the Internet.

There are several limitations to our study that, if seen from a different perspective, suggest improvements for future research. First and foremost, having a passive wait list group does not offer the best control for the effects of expectancy or adherence to the treatment schedule. However, we hypothesized that such a design would be suitable for examining the main effects of an Internet-delivered intervention in a new context. Future studies could compare the effects of Internet-delivered CBT and other interventions (such as, acceptance and commitment therapy) and explore whether one of these alternative treatments is more effective in reducing social anxiety symptoms. Second, only self-report measures will be used to assess participants’ symptoms in this study. Despite their many desirable traits (such as., simplicity, ease of administration, etcetera.), self-reports also display some limits (such as, responses based on the frail human memory and judgment, social desirability, responses influenced by question order, question wording, etcetera.) [48]. Therefore, future studies could also capture the underlying cognitive process by using the new generation of implicit measures that indirectly capture various psychological constructs. For example, the possible changes in participants’ automatic information processes occasioned by an online intervention could be investigated using the Implicit Association Test [49] or some other newly developed implicit measures. Finally, yet importantly, while Internet-delivered treatments have the advantage of increasing the accessibility of evidence-based interventions to a larger segment of the population, we suspect that the well-educated and computer savvy subgroup will mainly benefit from such programs. Alternative ways to increases treatment accessibility could be to offer straight forward, user-friendly programs that will not drive away beginners.

Overall, offering self-help materials with the support of new technologies constitutes a promising alternative for face-to-face treatments, and an effective way to disseminate evidence- based anxiety interventions. Using a predefined, and well-structured intervention that is available online decreases the number of therapist contact hours, and allows clinicians to assist a greater number of participants without sacrificing treatment efficacy. However, the costs and efforts needed for the development and maintenance of such specialized interventions, and for the appropriate training of new clinicians should not be underestimated. Because the initial costs for the platform development are relatively high, while the resources needed for maintenance and therapist contact hours are relatively low, it seems that once proven effective in an RCT, the optimal way of exploiting such platforms would be to attract a large number of clients. Considering the inverse ratio between the initial investment and the potential long-term benefits, it seems that using such platforms for a long time represents a cost-effective strategy worth pursuing.

## Trial status

The screening phase of this trial is planned for April and May, 2012.

## Competing interests

The authors declare that they have no competing interests.

## Authors' contributions

BTT initiated the study and made important contributions to the study design, and manuscript drafting. GA contributed to the study conception and design, and critically revised the manuscript. KNTM has been involved in drafting and revising the manuscript. All authors approved the final version of the manuscript.
